# Determination of antioxidant and antimicrobial activities of the extracts of aerial parts of *Portulaca quadrifida*

**DOI:** 10.1186/s13065-018-0514-2

**Published:** 2018-12-20

**Authors:** Zelalem Yibralign Desta, Desie Alemaw Cherie

**Affiliations:** 1 Department of Chemistry, College of Science, Bahir Dar University, P. O. Box 79, Bahir Dar, Ethiopia; 2 Agricultural and Nutritional Research Laboratory, Ethiopian Institute of Agricultural Research (EIAR), P.O Box 2003, Addis Ababa, Ethiopia

**Keywords:** *Portulaca quadrifida*, Total flavonoid content, Antioxidant activity, Total phenolic content and antibacterial activities

## Abstract

**Background:**

The extracts from the aerial parts of *Portulaca quadrifida* have been reported to show the total flavonoid content, antioxidant and antibacterial activities.

**Results:**

Our results revealed that the total flavonoid content of methanol and chloroform extracts is 2.335 ± 0.0097 and 1.7312 ± 0.0082 mgQE/100 g respectively. The two extracts also showed good antioxidant activity and total phenolic content as well as weak to moderate antibacterial activity against some bacteria.

**Conclusions:**

The extracts the aerial parts of the *P. quadrifida* showed good total flavonoid content, DPPH radical scavenging activity and antibacterial activity. In addition to this, the extracts also showed the presence of some important compounds by phytochemical analysis.
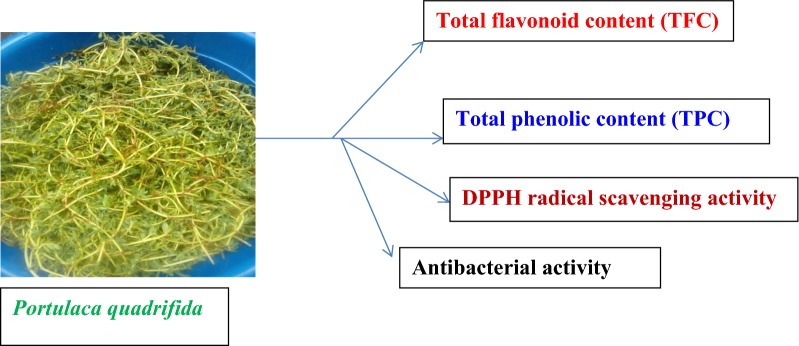

## Background

*Portulaca quadrifida*, commonly known as “chicken weed”, a herb belongs to the Portulacaceae family, is endemic to Ethiopia, in most dry area of Benshangul Gumuz, konso, Ethio-Somalia, Oromia, Kaffa and Hararghe regions. *Portulaca quadrifida* (known as “Kimma” in Amharic and Kawa in shinasha) is a prostrate, mat-forming annual or short-lived perennial herb with much branched, spreading, articulated, fleshy stems up to 50 cm long or longer, rooting freely from the nodes, often flushed reddish; nodes with a dense whorl of whitish hairs [1]. In Ethiopian traditional medicine, the aerial parts of *Portulaca quadrifida* used as food and used for the treatment of several diseases including gastric ulcer, ophthalmia, as an anti-microbial, anti-hyperglycemic, and for its antioxidant properties used in preventing different kinds of sickness and disorders [2]. Several studies suggested that *Portulaca quadrifida* is a good natural antioxidant that can be used as health promoting agent for various disorders including diabetes mellitus and other kinds of diseases [3]. Herein we reported the total flavonoid content, antioxidant activity and antibacterial activities of the extracts of the aerial parts of *Portulaca quadrifida*.

## Results and discussion

### Determinations of total phonolic content

The total phenolic content (TPC) of the extracts of the aerial parts of *P. quadrifida* was determined in terms of Gallic Acid equivalents per 100 g of dry weight of the sample (mgGA/100 g) as shown in Table 1. According to the study, it was evident that methanol extract had the highest level of phenolic content (4.9029 ± 0.0087 mgGAE/100 g) while petroleum ether extract of *P. quadrifida* was the least (2.4914 ± 0.0028 mgGAE/100 g). The maximum total phenolic content was recorded in more polar solvent extract whereas the minimum total phenolic content was recorded in non-polar solvent extract. These results suggested that the extraction of phenolic compounds is influenced by the polarity of the solvent used. In other ways, the reaction of Mo^6+^ (yellow), from Folin-Ciocalteu reagent, is changed to less oxidation state (Mo^+4^, Mo^+5^) (blue color) depend on the polarity of the solvent and the intensity of color indicates the concentration of phenolic content in the sample. Basically, methanol extract of *P. quadrifida* has better intense blue color, as compared to petroleum ether extract, which has very less color intensity. The total phenolic content result might vary by the maturity of the plant. The total phenolic content at the mature growth stage was lower than in the plants at the developing stage meaning levels of polyphenolic compounds decreased rapidly with age due to their dilution with leaf growth [4].

**Table 1:** Total phenolic content of *P. quadrifida* extracts

Different extracts	TPC in mgGAE/g dry extracts
Methanol extract	4.9029 ± 0.0087
Chloroform extract	4.5957 ± 0.0055
Petroleum ether extract	2.4914 ± 0.0028

Values are expressed as mean ± SD of triplicate analysis

### Determination of total flavonoid content

The total flavonoid content of the extracts of the aerial parts of *P. quadrifida* was evaluated and the results are shown in Table 2. The highest total flavonoid content of *P. quadrifida* extract was recorded in methanol extract (2.335 ± 0.0097) however the least total flavonoid content was recorded with petroleum ether extract (1.357 ± 0.0035). According to this study, the highest amount of the total flavonoid content was recorded in polar solvent extract and decreases with decreasing the polarity of the solvent in the same order as total phenolic content shown above.

**Table 2:** Total flavonoid content of the extracts of *P. quadrifida*

Different extracts	TFC in mgQE/g dray extracts
Methanol extract	2.335 ± 0.0097
Chloroform extract	1.7312 ± 0.0082
Petroleum ether extract	1.357 ± 0.0035

Values are mean of ± SD triplicate analysis

### Determination of antioxidant capacity

The antioxidant activity of the extracts of the aerial parts of *P. quadrifida* was evaluated by using DPPH radical scavenging assay as shown in Table 3. The scavenging effect of different extracts of *P. quadrifida* on the DPPH radical decreases in the order of methanol extract, chloroform extract and petroleum ether extract. The highest percentage inhibition for methanol extract compared to two other solvents could be due to high polarity nature of solvent and highly polar miscible compounds found in the plant. As the concentration of phenolic compound increases, degree of hydroxylation of the phenolic compound also increases which resulted to increase the scavenging activity (% inhibition). The increase in DPPH scavenging activity directly related to antioxidant capacity of the *P. quadrifida* extracts. As a result methanolic extract of *P. quadrifida* which has highest DPPH radical scavenging activity showed fairly highest antioxidant activity in comparison to other extracts. The presence of phenolic acid in the extract of tested plant increases the probability of reaction with free radical, which lead to decrease the amount of free radical. In this study antioxidant assay of DPPH radical scavenging capacity (%), total phenolic, and total flavonoid contents were used to evaluate the antioxidant activity of *P. quadrifida* extracts. A good correlation between DPPH radical scavenging activity with TPC (R^2^ = 0.782) was observed and furthermore, a reasonable correlation between DPPH and TFC (R^2^ = 0.996) was observed. Results obtained from the experimental data showed that there was good correlation between total flavonoid and antioxidant activity of different extracts of *P. quadrifida* with correlation coefficient (R^2^) 0.996.

**Table 3:** DPPH radical scavenging activity of *P. quadrifida* extracts at different concentrations

S. no	Concentration of extract (mg /L)	%DPPH scavenging activity		
		Methanol	Chloroform	Petroleum ether
1	20 (w/v)	25.3541 ± 0.0272	23.1373 ± 0.00804	22.8602 ± 0.0020
2	40 (w/v)	30.9421 ± 0.0038	29.4874 ± 0.0100	26.4547 ± 0.0188
3	60 (w/v)	36.8042 ± 0.0056	34.1595 ± 0.0054	30.8344 ± 0.0047
4	80 (w/v)	38.2620 ± 0.0003	37.3599 ± 0.0062	33.0511 ± 0.0039
5	100 (w/v)	46.7595 ± 0.0069	42.4030 ± 0.0053	44.5428 ± 0.0174

### Determination of antimicrobial activity of *P. quadrifida*

Different extracts from the aerial parts of *P. quadrifida* demonstrated antibacterial activities against both Gram-positive and Gram-negative bacteria strains. In this study 250 mg/L and 200 mg/L of methanol extract of *Portulaca quadrifida* recorded the most significant antimicrobial activity against all tested bacteria but chloroform extract showed good activity against fungi. Though methanol extract showed broad spectrum of activity against all tested bacteria, especially *S. aurues* and *E. coli* are the most susceptible pathogens. In addition, the methanol extracts of *Portulaca quadrifida* also showed significant antibacterial activity against Gram negative bacteria (*Escherichia coli* and *Klebsiella pneumoniae*) and Gram positive bacteria (*Staphylococcus pyogenes and Staphylococcus aureus*), respectively. However, petroleum ether extract showed least activity against all bacterial organisms. The significant and higher antibacterial activity might be due to the presence of flavonoids in the plant as described by Mutyala and Kishore [5]. 0.3% w/v of Gentamycine drug reference and DMSO were used as a positive and negative control respectively. Gentamycine showed 27, 17, 29 and 19 mm zone of inhibition on *E. coli, K. pneumoniae*, *S. aureus* and *S. pyogenes* respectively while DMSO had no sensitivity on those four bacterial species.

### Determination of anti-fungal activity of the extracts of *P. quadrifida*

In this study the drug reference which was used for antifungal test was Gentamicine (0.3% w/v) as a positive control and DMSO as a negative control. Gentamycine has 23 and 18 mm zone of inhibition on *yeast* and *mold* respectively but DMSO has no activity on the above two fungal species. As shown in Tables 4 and 5, each different extracts of the aerial parts of *P. quadrifida* showed a good inhibition zone against each bacterium and fungus species at 250 mg/mL and 200 mg/mL of extract. The antifungal activity of chloroform extract of *P. quadrifida* dominates over methanol and petroleum ether extracts towards the tested pathogens. It indicates, either the microbes might be relatively resistant to compound of the plant which is extracted by methanol and petroleum ether. The result of this study has shown that all the isolated bacteria were susceptible to all solvent extract of *P. quadrifida* in agar well diffusion assay. Thus *P. quadrifida* extracts will provide good ways to control microbial infection especially caused by those multidrug-resistant pathogens. Although the most different extracts of *P. quadrifida* showed lower activities compared to the controlled used during the study. It has promising result that the plant has antimicrobial substance with an optimum potential to inhibit growth of tested pathogens in advance over clinically used antibiotics compared with cost and side effect.

**Table 4:** Antibacterial sensitivity of *Portulaca quadrifida* extracts

Test bacteria	Concentration in (mg/L)	Mean zone of inhibition ± SD (mm)		
		Petroleum ether	Chloroform	Methanol
*Escherchia coli*	250	7.1 ± 1.6	8.0 ± 2.0	11.88 ± 1.0
	200	5.3 ± 1.3	4.0 ± 0.7	9.7 ± 0.8
	150	1.2 ± 0.8	1.8 ± 2.6	6.4 ± 1.3
	100	Growth	Growth	3.8 ± 1.7
	50	Growth	Growth	Growth
*Klebsiella pneumoniae*	250	5.3 ± 1.1	5.78 ± 0.5	10.3 ± 1.3
	200	2.5 ± 0.6	3.1 ± 2.1	5.2 ± 0.6
	150	Growth	Growth	1.5 ± 1.4
	100	Growth	Growth	Growth
	50	Growth	Growth	Growth
*Staphylococcus aureus*	250	10.3 ± 2.2	10.7 ± 3.0	17.3 ± 2.0
	200	6.2 ± 2.1	6.8 ± 2.5	10.2 ± 1.75
	150	1.2 ± 1.0	1.5 ± 0.7	5.3 ± 0.6
	100	Growth	1.2 ± 0.5	2.35 ± 0.5
	50	Growth	Growth	Growth
*Streptococcus pyogenes*	250	8.7 ± 1.7	9.3 ± 2.1	10.8 ± 2.5
	200	5.0 ± 1.3	6.8 ± 1.2	7.3 ± 1.8
	150	2.9 ± 1.8	1.4 ± 2.3	3.0 ± 2.5
	100	Growth	Growth	Growth
	50	Growth	Growth	Growth

Values are expressed in mean ± SD of three individual experiments

The word “growth” represents zero inhibition zone the extract at a given concentration

**Table 5:** Inhibition zone of *P. quadrifida* extracts against standard fungus

Test fungi	Concentration in (mg/L)	Mean zone of inhibition ± SD (mm)		
		Petroleum ether	Chloroform	Methanol
Yeast	250	8.4 ± 0.6	11.8 ± 1.6	10.2 ± 2.1
	200	4.3 ± 0.7	6.0 ± 2.2	7.2 ± 2.0
	150	Growth	2.4 ± 2.5	3.0 ± 1.3
	100	Growth	Growth	1.2 ± 1.0
	50	Growth	Growth	Growth
Mold	250	12.5 ± 2.1	15.2 ± 1.5	10.5 ± 1.6
	200	8.3 ± 1.3	7.7 ± 1.7	9.7 ± 1.5
	150	3.3 ± 0.8	3.0 ± 0.7	4.3 ± 2.1
	100	Growth	1.2 ± 1.7	Growth
	50	Growth	Growth	Growth

Values are expressed in mean ± SD of 3 individual experiments

The word “growth” represents zero inhibition zone the extract at a given concentration

### Estimation of acute toxicity

As shown in Table 6 below, aerial parts of *P. quadrifida* extracts were tested on laboratory animals under 14 days of observation period. None of the animals were showed any negative sign, depression, and symptom during observation period as compared with control animals. The result was showed a positive correlation between control and experimental animals regarding to the weight and other physical features of animals. Principally, the assumptions of Mutyala and Kishore [5] and Burkill [6] are not agreed with the outcomes of the present study.

**Table 6:** Experimental weight of animals that were recorded within fixed time interval

Day	Animal weight (g)	Mice 1 in gram	Mice 2 in gram	Mice 3 in gram	Mice 4 in gram
Day 1	Test animal	35.500	37.420	38.140	39.180
	Control animal	35.160	35.860	37.450	39.380
Day 7	Test animal	37.640	39.500	40.240	41.850
	Control animal	37.880	36.510	37.940	41.500
Day 14	Test animal	39.240	41.180	43.500	43.880
	Control animal	39.000	39.250	40.450	43.850

## Experimental

### Plant materials

The fresh aerial parts of *Portulaca quadrifida* were collected in September 2017 from Benishangul Gumuz Regional State, Bullen district, Western Ethiopia, which is 310 km away from Bahir Dar and 680 km from Addis Ababa. The plant species was identified and authenticated by Dr Ali Seid at Biology department, Bahir Dar University.

### Chemicals and reagents

The analytical grade chemicals and reagents used for this study were petroleum ether (Blulux, india), chloroform (Lobachemie, India), methanol (Lobachemie, India), dimethyl sulphoxide (DMSO), potassium iodide (Lobachemie, India), Wagner’s reagent (Iodine in potassium iodide), aluminum chloride (Blulux, India), iodine (Blulux, India), sodium nitrite, hydrochloric acid (LOBAChemie, India), sulfuric acid (Lobachemie, India), sodium hydroxide (Mumbai-400002, India), nitric acid, sodium carbonate, disodium hydrophosphate (Blulux, India), phosphoric acid (Blulux, India), sodium molybdate, sodium tungstate, iron chloride (Alpha Chemica, India), bromine, ascorbic acid (Blulux, INDIA-121005), Gallic acid, DPPH (himedia, India), quercitine (Alpha chemika, India), lithium sulphate, Muller Hinton agar and ammonia solution. All chemicals used for laboratory analysis were analytical grade that is greater than 97% in purity.

### Extraction and isolation

The air-dried and ground aerial parts of *P. quadrifida* (200 g) were extracted by soaking successively in petroleum ether, chloroform (CHCl_3_) and methanol (MeOH) each for 48 h (two times with each solvent) and removal of the solvent under reduced pressure using a BUCHI flash evaporator to afford extracts of 2.70 g (for petroleum ether), 7.30 g (for chloroform) and 8.26 g (for MeOH).

### Determination of total phenolic content (TPC)

Total phenolic content of the aerial parts of *P. quadrifida* extracts was determined according to the Folin-ciocalteu method as described before by different researchers [7, 8]. Each extract was dissolved in methanol (100 g/L) and 1 mL of extract, 5 mL of Folin-Ciocalteu reagent (diluted tenfold) and 4 mL (75 g/L) of sodium carbonate (Na_2_CO_3_) solution were added together. After the reagents mixed with the extracts, the flasks were filled with distilled water up to the mark and the mixtures left for 30 min in dark area and absorbances were measured at 765 nm.

### Determination of the total flavonoid content (TFC)

The total flavonoid content of *P. quadrifida* crude extracts were determined by aluminum chloride assay as described before by different researchers [9, 10]. 0.25 mL of the extract was mixed with 1.25 mL of distilled water in 50 mL volumetric flask, followed by an immediate addition of 0.075 mL of 5% NaNO_2_ and 5 min later, 0.15 mL of 10% AlCl_3_ solution was added. After 6 min 0.5 mL of 1 M NaOH solution was added followed by 0.275 mL of distilled water and immediately the absorption at 510 nm was recorded by using UV-Vis spectrophotometer.

### Free radical scavenging activity

Free radical scavenging activity of the extracts was determined by using the DPPH assay. DPPH assay is popular in natural product antioxidant studies and the antioxidant activity of *P. quadrifida* extracts was also evaluated on the bases of the radical scavenging effect of the stable 2,2-diphenyl-1-picrylhydrazyl (DPPH) by using those methods described previously by different researchers [11]. The methanol, chloroform, and petroleum ether extracts of *P. quadrifida* with different concentrations (20%, 40%, 60%, 80% and 100% (v/v)) were prepared by methanol to determine DPPH scavenging activity. An aliquot of 2 mL of 0.004% of DPPH solution was mixed with 1 mL of each extracts and the solutions were kept in dark for 30 min and the absorbance of the combination was measured at 517 nm using UV–Vis spectrophotometer.

### Antimicrobial activity test

Antimicrobial activities of the plant extracts *P. quadrifida* were done in microbiology laboratory, department of Biology at Bahir Dar University by using agar well diffusion method. Muller Hinton agar media was prepared for culturing selected Gram negative and positive bacteria by using standard methods. Four bacteria were selected, two Gram positive (*S. aureus and S. pyogenes*) and two Gram negative (*E. coli*, *and K. pneumoniae*). A series of plant extracts with concentrations (50, 100, 150, 200 and 250 mg/L) and standard antibiotics (*Gentamycin*) were added to the incubated plate by using filter paper. Then it was incubated for 24 h at 37 °c and the experiment was repeated thrice, and the average values of zone of inhibition was recorded in mm for antimicrobial activity [12–14].

### Estimation of cytotoxicity

The toxicity of the plant was done at Ethiopian food, medicine and health care administration and control authority. Experiments were performed using healthy young adult mice, non-pregnant and weighing 35–40 g. The experimental animals were divided into control and test groups containing four animals each. The animals were grouped in to their order of age and feed the plant for experimental animals and pellet for control group. Young rats were chosen because of their greater sensitivity to treatment. All the rats were observed individually at least once during the first 30 min, periodically during the first 24 h with special attention given during the first 4 h, and then daily for a total of 14 days. All the rats were observed at least twice daily with the purpose of recording any symptoms of ill-health or behavioral changes [15].

### Methods of data analysis

#### Analytical equations

In this study, the antioxidant activity, total phenolic content and total flavonoid content of *P. quadrifida* extracts were calculated and reported in terms of ascorbic acid (AA), gallic acid (GA), and querecetin (QT) equivalent per gram of extract. The equation stated below used for calculation from Y = BR + C linear equation.

$${\text{W}}\left( {\frac{mg}{g\,of\,extract}} \right) = \frac{{ R\left( {mg/mL} \right)\left( {volume\,of\,extract\left( {mL} \right)} \right)}}{weight\, of\,dry\,sample\,in\,gram}$$

where: W = GA, AAE, QT ${{{\text{R }}\left( {{\text{mg}}/{\text{mL}}} \right) \, = \frac{Y - C}{B}} \mathord{\left/ {\vphantom {{{\text{R }}\left( {{\text{mg}}/{\text{mL}}} \right) \, = \frac{Y - C}{B}} {1000}}} \right. \kern-0pt} {1000}}$, Y = absorbance of the sample, C = y-intercept from calibration curve and B = slope from calibration curve.

The percentage of DPPH radical scavenging activities of *Portulaca quadrifida* extract was calculated with equation stated below:

$${\text{DPPH radical scavenging}}\left( \% \right){\text{ activity }} = \, \left[ {\frac{{\left( {Ao - A1} \right)}}{Ao}} \right] \, \times { 1}00$$

where: A_0_ = absorbance of the control, A_1_ = absorbance of the sample.

### Data analysis

Total phenolic content, total flavonoid content, antioxidant activities in DPPH assay and zone of inhibition in antimicrobial activities were measured in triplicates to take the mean ± SD value. The calibration curves and graphs were constructed by using Microsoft excel 2007. Statistical analysis was also undertaken by analysis of variance (one way ANOVA) with Least Significant Difference (LSD) to compare result between extracted plants by different solvents at the same concentrations using SPSS statistics version 20. Result was considered statistically significant at P-value < 0.05.

## Conclusion

The extracts of the aerial parts of the *Portulaca quadrifida* were subjected to qualitative and quantitative analysis. The result of the study clearly indicated that nearly all investigated extracts showed antioxidant activity against DPPH radical scavenging activity. In antioxidant activity measurement, methanol extract, showed the highest percentage of DPPH radical scavenging activity. In addition to this, the study also showed that *P. quadrifida* extracts were found to contain measureable amount of total phenolic and flavonoid content which play major role in inhibiting oxidative stress in the body. Moreover, the results can also provide the effectiveness of *P. quadrifida* extracts for antimicrobial activity. All extracts of the plant also showed antimicrobial activity against different bacteria and fungi species. The acute toxicity result also revealed that the plant has no observable side effects. Generally the result of the study showed that the plant contains significant secondary metabolites and can be used as easily accessible source of food, natural antioxidant and antimicrobial activity.
